# Detection of Volatile Organic Compounds (VOCs) in Urine via Gas Chromatography-Mass Spectrometry QTOF to Differentiate Between Localized and Metastatic Models of Breast Cancer

**DOI:** 10.1038/s41598-019-38920-0

**Published:** 2019-02-21

**Authors:** Mark Woollam, Meghana Teli, Paula Angarita-Rivera, Shengzhi Liu, Amanda P. Siegel, Hiroki Yokota, Mangilal Agarwal

**Affiliations:** 1 IUPUI, Department of Chemistry and Chemical Biology, Indianapolis, 46202 USA; 2 IUPUI, Department of Biomedical Engineering, Indianapolis, 46202 USA; 3 IUPUI, Department of Mechanical Engineering and Energy, Indianapolis, 46202 USA; 4 Integrated Nanosystems Development Institute, Indianapolis, 46202 USA; 5 Biomechanics and Biomaterials Research Center, Indianapolis, 46202 USA

## Abstract

Breast cancer is the most common cancer detected in women and current screening methods for the disease are not sensitive. Volatile organic compounds (VOCs) include endogenous metabolites that provide information about health and disease which might be useful to develop a better screening method for breast cancer. The goal of this study was to classify mice with and without tumors and compare tumors localized to the mammary pad and tumor cells injected into the iliac artery by differences in VOCs in urine. After 4T1.2 tumor cells were injected into BALB/c mice either in the mammary pad or into the iliac artery, urine was collected, VOCs from urine headspace were concentrated by solid phase microextraction and results were analyzed by gas chromatography-mass spectrometry quadrupole time-of-flight. Multivariate and univariate statistical analyses were employed to find potential biomarkers for breast cancer and metastatic breast cancer in mice models. A set of six VOCs classified mice with and without tumors with an area under the receiver operator characteristic (ROC AUC) of 0.98 (95% confidence interval [0.85, 1.00]) via five-fold cross validation. Classification of mice with tumors in the mammary pad and iliac artery was executed utilizing a different set of six VOCs, with a ROC AUC of 0.96 (95% confidence interval [0.75, 1.00]).

## Introduction

Breast cancer is the most commonly diagnosed cancer among all women worldwide, but there is no accurate and non-invasive method to screen for breast cancer in patients before a confirmatory biopsy is performed^1^. Implementing an accurate and non-invasive screening technique is important because the earlier that a cancerous tumor is found in the human body, the more efficient treatment will be^2^. The current non-invasive screening methods that are used to screen for breast cancer include mammography and ultrasounds, but these screening techniques are not sensitive or specific, which leads to many false positive results. Overall, these methods lead to over-diagnosis and over-treatment^3^. Another non-invasive screening method that can be used to screen for breast cancer is detecting hypermethylation of DNA in nipple aspirate fluid^4^, but sample collection poses a challenge. Urine contains volatile organic compounds (VOCs) that are products of metabolic pathways and may serve as a source of biomarkers for breast cancer^5,6^. VOC biomarker discovery is promising because there are thousands of VOCs that are present in urine, breath and blood samples that have the potential to be biomarkers for an array of diseases^7,8^. The detection of VOCs has been a recent alternative screening technique for many different diseases that has been shown to be sensitive and specific. Analyzing urine samples for metabolic biomarkers is also relatively inexpensive compared to other traditional techniques^3^. Implementing a non-invasive and accurate breast cancer diagnostic technique based on sensing metabolic VOCs associated with the disease can lead to an increase in early diagnosis^9^.

An alternative sample that could be collected to analyze VOCs as potential biomarkers for breast cancer is biological breath^10–13^. Phillips *et al*. discovered a set of VOCs found in human breath that distinguished between patients with and without breast cancer with 78.5% sensitivity and 84.8% specificity in their training data set^10^. Even though some cancer VOC biomarkers have been identified in human breath, analyzing urine can provide better insight into metabolic biomarkers. For example, urine has relatively higher concentrations of metabolic VOCs than breath, which makes them easier to detect^14^. Analysis of cell line VOCs is another technique utilized to discover biomarkers related to breast cancer^3,15^. Silva *et al*. (who previously analyzed human urine VOCs)^14^ reported a set of VOCs that distinguish between breast cancer and healthy cultured cells. One-way ANOVA initially identified VOCs statistically significantly different between healthy and breast cancer cell lines, and then Principal Component Analysis (PCA) and Linear Discriminant Analysis (LDA) were utilized to classify cell lines using multiple compounds^15^. Even though analyzing cell lines is an efficient strategy, these results may not be translatable to human or even whole animal studies. Analyzing urine would provide biomarkers that change not only because of transformation of tumor cells, but also changes in tumor local microenvironment. This property, for example, may play a role in the transition of some adenocarcinomas from ductal carcinoma *in situ* (DCIS) to invasive^16^. This can be studied by identifying VOCs in mouse urine that are associated with tumors in the mammary pad compared with the same tumors injected to the bone. The only publications that analyze mouse urine to discover VOC biomarkers identified metabolic trends in lung cancer^17–19^.

Metabolic biomarkers, including VOC biomarkers, are generally reported as panels or signatures of compounds rather than individual metabolites. A panel can better identify trends and multivariate analysis can be cross validated for accuracy better than a single metabolite^20^. There are pitfalls, however, which can occur when building a multivariate model. For linear models, there is the potential problem of multicollinearity^21^, but all models could be unstable or overfit^22,23^. Utilizing overfit models is problematic because the accuracy of classification will decrease when implemented on an independent data set. Data and function perturbation are two techniques used to detect overfit models^23^.

Solid phase microextraction (SPME) coupled to gas chromatography-mass spectrometry (GC-MS) is widely used for VOC biomarker discovery^24,25^. SPME utilizes a silica-based fiber to which the VOCs in the headspace of the sample adsorb when the urine is heated and agitated. After incubating, the fiber is injected directly in to the GC-MS system where the front inlet is kept at a relatively high temperature, and the VOCs thermally desorb off the fiber and enter the chromatographic column where they can be separated and identified^26^. Analyzing mouse urine via SPME coupled to GC-MS as a pilot study can provide information on urinary VOCs that classify breast cancer from no cancer and metastasized breast cancer from localized. Also, discovering biomarkers in a simplified biological model where the conditions of the experiment can be controlled makes it easier to find endogenous metabolic biomarkers. One problem which occurs when using GC-MS to analyze mouse urine is that the urine contains major urinary proteins (MUPs) that have hydrophobic pockets where VOCs preferentially bind. Therefore, the MUPs must be denatured so the VOCs can be released into the sample headspace and analyzed via GC-MS^27^. Guanidine hydrochloride (GHCl) is a well-known reagent that both denatures the MUPs significantly and increases the ionic strength of the sample solution which also increases the concentration of volatiles in the headspace of the sample^28^. Herein, mouse urine samples were analyzed via SPME coupled to GC-MS quadrupole time-of-flight (QTOF) to differentiate two different locations of mammary tumors and samples without tumor injection based solely on VOC composition.

## Methods

### Materials and Instrumentation

All BALB/c female mice utilized during the study were purchased from Harlan Laboratories, Indianapolis, IN, USA. 4T1.2 mammary tumor cells were attained from Dr. R. Anderson at the Peter MacCallum Cancer Institute in Melborne, Australia. Two cm PolyDimethylMethylSiloxane/CARboxen/DiVinylBenzene (PDMS/CAR/DVB) SPME fibers manufactured by Supelco were purchased from Sigma Aldrich, and 10 mL headspace vials as well as 18 mm magnetic lids with a screw thread cap from Restek. Eight Molar Guanidine Hydrochloride (pH = 8.5) was purchased from Sigma Aldrich. An Agilent 7890 A GC system coupled to an Agilent 7200 Accurate-Mass Quadrupole Time-of-Flight MS system with a front-end PAL autosampling system (CTC Analytics) was utilized to incubate samples and separate/identify VOCs. The column employed was an Agilent HP-5ms, 5% phenylmethyl siloxane GC column of 30 meters in length, 250 micrometer internal diameter and 0.25 micrometer film thickness.

### Mouse Urine Collection

Female BALB/c mice were kept in cages and fed the same diet to limit metabolic variations due to nutrition. All of the procedures conducted during this experiment were approved by Indiana University Animal Care and Use Committee. All experimental procedures followed the Guiding Principles in the Care and Use of Animals that is supported by the American Physiological Society. 4T1.2 tumor cells were cultured in Dulbecco’s Modified Eagle Media (DMEM). The BALB/c mice were injected in the mammary pad with 4T1.2 mammary tumor cells to represent localized cancer. The same cells were injected in the iliac artery of a different group of mice to model metastasized breast cancer. Mice not injected with any tumor cells served as a control. Mice injected with mammary tumors in either location will be referred to as mice with breast cancer, mice with mammary pad tumors as localized and mice with tumors injected in the iliac artery as metastasized breast cancer. Injection into the iliac artery is an accepted model of metastasized cancer^29^. Bone is a common region where breast cancer metastasizes to because of the high affinity for bone that breast cancer cells exhibit^30,31^. The localized and metastasized tumor models were previously used and justified in literature^29^.

Urine was collected 18 days after the mice were injected. No visual signs of injury due to injection were observed when the urine was collected. Samples were collected (approximately 75 microliters) in two time periods, with the first time period collecting urine from control, mammary pad and metastasized cancer mice and the second urine from control and metastasized cancer mice. Mice are moved to a cage where the floor has been covered in fresh parafilm. Urine falls on the parafilm and is collected using pre-cleaned glass Pasteur pipettes into pre-cleaned glass headspace vials which were put on dry ice immediately. All the mouse urine samples were stored in a −80 °C freezer in a 10 mL headspace vial before analysis. All urine was collected in the morning to avoid and limit variation due to void times. One hour before agitation and extraction, eight M GHCl was added in a one to one ratio to denature the MUPs and increase the ionic strength of the sample solution.

### SPME and GC-MS QTOF

The VOCs were captured by incubating a pre-conditioned SPME fiber in urine headspace before analysis. SPME fibers were conditioned every day for ten minutes prior to the first run, and for four minutes after each run. Mouse urine samples in headspace vials were agitated and heated to 60 °C for a total of 30 minutes. Next, the SPME fiber was placed inside the headspace of vial through the septum for a total of 30 minutes while the sample continued agitating and heating at 60 °C. After extraction, the SPME fiber was injected into the inlet of the GC-MS QTOF at 250 °C while the mass transfer line was held at 230 °C. The oven temperature program implemented consisted of holding the temperature at 40 °C for the first 2 minutes of the chromatographic run. After, the temperature was ramped to 100 °C at a rate of 8 °C/min, followed by a 15 °C/min ramp to 120 °C, 8 °C/min to 180 °C, 15 °C/min to 200 °C and finally an 8 °C/min ramp to 260 °C. Data was collected utilizing Agilent Chemstation software. Parameters utilized for SPME coupled to GC-MS QTOF were previously optimized, including: SPME fiber coating, agitation time, extraction time, agitation and extraction temperature, and volume of sample. Due to the limited amount of urine collected from each mouse (<100 microliters), only one injection into the GC-MS system was conducted per sample.

Reproducibility of extraction procedure was tested as follows. High-density polyethylene (HDPE) virgin pellets generate a consistent and complex matrix of VOCs that does not degrade substantially over time. In order to quantify reproducibility of the SPME extraction procedure, HDPE pellets were run on five consecutive days. The relative standard deviation (RSD) of the total integrated signals was 1.17%. Six representative VOCs conserved across samples (saturated and unsaturated hydrocarbons off-gassed by the HDPE pellets) were selected to observe the reproducibility of the integrated signal over five consecutive days, and the RSD values were below 6% (range of 1.1–5.5%) for each of the six volatiles.

### Data Screening and Analysis

Mass Hunter Quantitative Profinder was utilized to spectrally align multiple chromatographic peaks obtained from all samples using similarities in experimental retention time and mass spectrum. Profinder generates a matrix that includes all the retention times and integrated signals for every VOC in each sample. The log2 of the integrated signal values were calculated to transform the data matrix to an approximate Gaussian distribution^32–34^. Compounds were filtered by requiring either a two-tail Student’s T-test or Wilcoxon’s Rank sum test p-value < 0.1. While not all of these compounds have an alpha <0.05, they may still have utility at constructing a multiparametric test. In addition, p-values obtained from univariate statistical analysis were not corrected for multiple testing. Univariate methods were used to screen for VOCs that might be useful for multivariate analysis, where statistical significance can be measured through model stability testing including cross-validation, bootstrapping, and method perturbation. Multivariate tests can, if properly validated, utilize univariate compounds with broader confidence intervals^20^. Normality of the data was not tested, therefore, both a parametric and non-parametric test were employed to find statistically significant features. Individual VOCs that had high within class variation (collected from the two different time periods described above) were removed from the sample matrix as likely environmentally based differences. Hierarchical heatmaps were generated for both comparisons by z-scoring all log2 integrated signal values for all VOCs detected in every sample. The hierarchical heatmap was generated using a Euclidean distance metric and average linkage to generate the hierarchical tree (Matlab). VOCs are sorted in the hierarchical heatmap on the y-axis by similarities in concentration among the samples that were analyzed. PCA was used for visualization of patterns and outliers (no samples removed as outliers). Iterative LDA^35^, a forward selection method in which features are selected for their ability to discriminate between data sets^22^, was executed on a matrix composed of the compounds identified by univariate analysis. The combination of VOCS that produced the highest area under the receiver operating characteristic (ROC) curve generated via LDA were also tested via five-fold cross validation (Matlab) to test if the model is overfit (data perturbation)^36^. Five-fold cross validation was performed 500 times to produce an estimated ROC value. A 95% confidence interval for the area under the ROC associated with five-fold cross validation was obtained by bootstrapping the results 500 times with randomly selected samples^37^. Function perturbation was performed on the developed test matrix by implementing a logistic regression classification algorithm in Matlab to further test if the models are overfit^23^. In addition, the two test matrices of VOCs were tested for multicollinearity^21^ by performing linear regression in Matlab on the predictor and response variables. The Variable Inflation Factor (VIF) was measured to assess the degree of multicollinearity in the two models (cancer/no cancer and localized/metastatic). A VIF threshold of 10 demonstrates a strong correlation between predictor values^38^. Iterative LDA was also performed on the same set of data to distinguish between all three classes of samples.

### Identification of metabolites

All VOCs that were found as p < 0.1 via the Student’s T-test or Wilcoxon’s Rank sum test in both data sets and had low within class variation were identified utilizing Mass Hunter Quantitative Profinder, Mass Hunter Unknown Analysis and the NIST14 mass spectral library. NIST14 was uploaded to Unknown Analysis, and sample chromatograms were deconvoluted and all the features were identified. The retention time and mass spectrum produced from Profinder  were used to find the corresponding feature in Unknown Analysis. If the retention time/mass spectrum matched, and there was a match factor higher than 65, the compound was identified. To confirm that identification was correct, the non-polar retention index (NPRI) from NIST was compared to the experimental NPRI calculated from the average retention time of the feature. If the NIST and experimental NPRI values were within 100 units, the compound was deemed identified. Pure chemical compounds were not purchased or analyzed by GC-MS QTOF to confirm the identification of VOC biomarkers. The Human Metabolomic Database (HMDB) was utilized to identify compounds that were endogenous to the human body, on the assumption that such metabolites were likely also endogenous to mice. VOCs that were not found on HMDB were included in the sample matrix: likely excreted compounds that were not in HMDB were murine-specific and endogenous, bacterial in origin, or food source related.

## Results

### Urine Sample Collection

Urine was collected from 12 mice with no cancer, eight mice with mammary pad cancer and 22 mice with metastasized cancer. Of the 42 mice, analysis was only performed on urine samples from 36 mice because samples from six of the mice, there was less than 75 microliters present (11 no cancer, eight localized and 17 metastasized mouse urine samples had enough urine for processing).

### Univariate Statistical Analysis and Compound Identification

**Figure 1:**
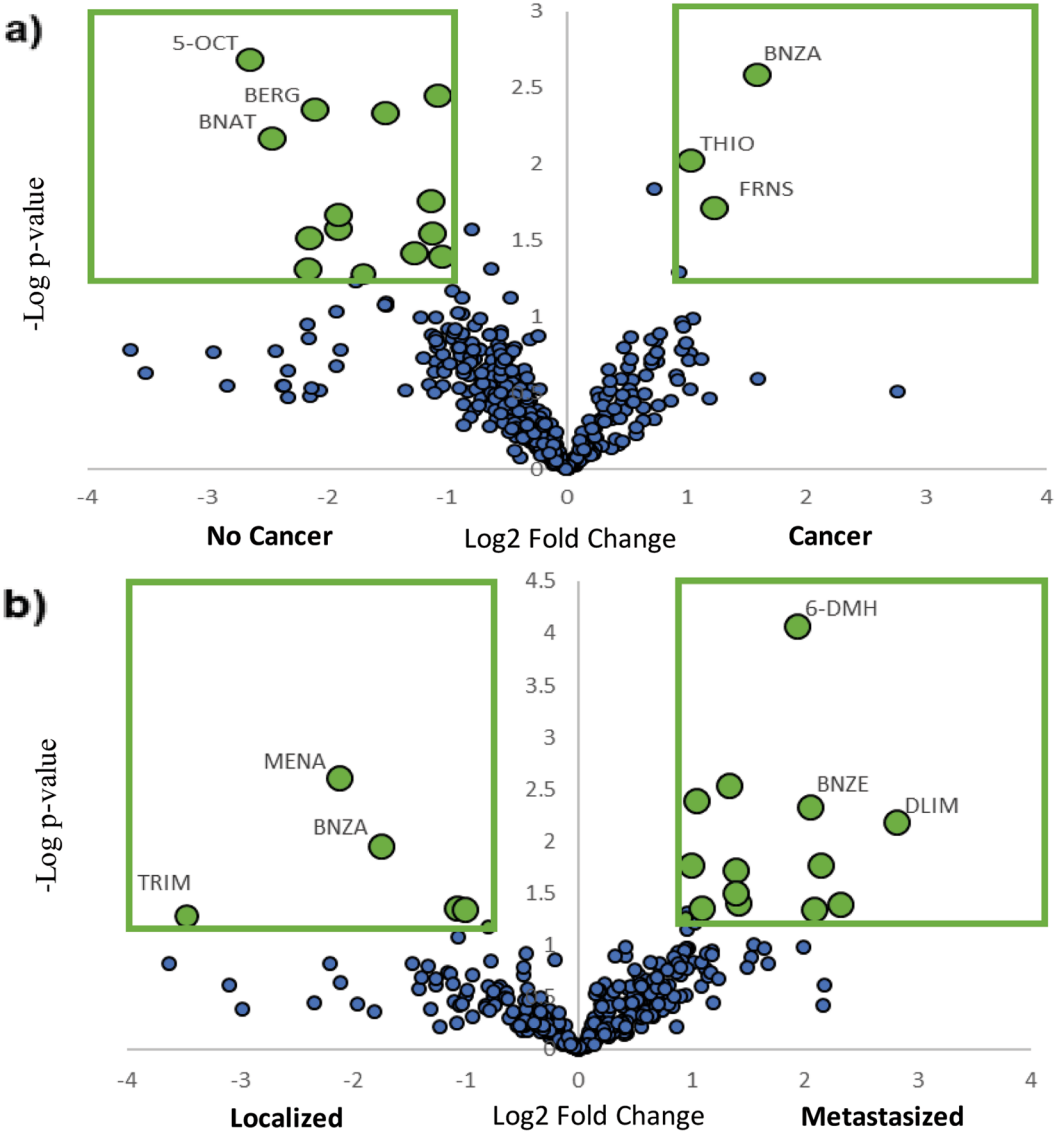
(**a**) Volcano plot where statistical significance via the Student’s T-test is plotted against log 2-Fold Change between classes for metabolites present in at least half of one class, distinguishing between mouse urine with and without cancer (5-OCT = 5-Octen-1-ol, BERG = Bergamotene, BNAT = Benzeneacetaldehyde, BNZA = Benzaldehyde, THIO = Thiophene, 2-pentyl, FRNS = Farnesene), (**b**) Volcano plot in a similar fashion produced to distinguish between mouse urine with localized and metastasized breast cancer (MENA = Menadione, TRIM = 2,6,6-Trimethyl-2-cyclohexene-1,4-dione, 6-DMH = 6,6-Dimethylhepta-2,4-diene, BNZE = Benzene, 4-ethenyl-1,2-dimethyl-, DLIM = D-Limonene).

To answer the question of which VOCs have high discriminating power to distinguish between cancer/no cancer and localized/metastasized, all 36 samples were spectrally aligned utilizing Profinder. For cancer (n = 25)/no cancer (n = 11), this alignment produced 646 compounds detected in at least half of one of the two sample classes. For mammary pad (n = 8) and metastatic (n = 17) samples, 601 compounds were present in at least half of one of the two classes. Univariate statistical analysis showed that there were 226 features that could distinguish between mice with cancer and no cancer (p-value < 0.1 by Student’s t-test or Wilcoxon Rank sum). On the other hand, only 125 compounds were different between localized and metastasized breast cancer urine samples collected from the mice (p < 0.1). Figure 1 shows the volcano plots for the two sets. For both volcano plots, the VOCs that are highlighted and outlined in green have an absolute log 2-Fold Change value greater than one, and their p-value produced from the Student’s T-test < 0.05. Metabolites that have a positive log 2-Fold Change value are up regulated in breast cancer or metastatic cancer and metabolites with negative values are down regulated. In the cancer/no cancer volcano plot, there are 17 metabolites that meet the required statistical criteria. Out of the 17 metabolites highlighted in green, 14 VOCs are down regulated in breast cancer and there is a total of three VOCs which are up regulated. In the volcano plot for VOCs classifying localized and metastasized cancer, there are 18 metabolites that meet the statistical criteria; 13 of the 18 metabolites which meet the criteria are up regulated in metastasized breast cancer and five are down regulated. In both volcano plots, six VOCs (three that are up regulated and three that are down regulated) with the lowest p-values and highest absolute log 2-Fold Change values are labeled utilizing their abbreviations which can be seen in Tables 1 and 2. Out of the VOCs that are labeled in both plots, Benzaldehyde (BNZA) is the only VOC that can be observed in both volcano plots. Of the 226 features that were univariately different (p < 0.1) between mice with and without breast cancer, 43 VOCs (identified by mass spectrum) had low within class variation (means of results from time period one and time period two comparable). Similarly, of the 125 VOCs that univariately distinguished between mice with breast cancer in the mammary pad and metastasized to the bone, 30 had low within class variation.

**Table 1:** List of the 43 VOCs that have a p-value less than 0.1 via the Student’s T-test or Wilcoxon’s Rank sum test when classifying mice with no cancer and mice that have breast cancer.

Name	Abbrev.	RT (min)	T test p-value	Rank-sum p-value	Regulation	CAS #
5-Octen-1-ol, (Z)-*	5-OCT	8.6	2.3E-4	1.4E-4	down	64275-73-6
Benzene, 4-ethenyl- 1,2-dimethyl-* ^**,ǂ**^	**BNZE**	**10.45**	**4.5E-4**	**6.3E-4**	**down**	**27831-13-6**
Bicyclo[3.1.0] hexan-2-one, 3,3,6-trimethyl-*^,ǂ^	BCY3	8.87	0.003	7.6E-4	down	53966-40-8
Bicyclo[2.2.1]heptane, 7,7-dimethyl-2-methylene* ^**,ǂ**^	**BCY2**	**8.52**	**0.006**	**0.006**	**down**	**471-84-1**
Pinocarvone* ^**,ǂ**^	**PINC**	**11.66**	**0.017**	**0.002**	**down**	**30460-92-5**
Benzyl methyl disulfide*	BMDS	15.07	0.076	0.052	down	699-10-5
Benzene, 1-ethyl-4-methoxy-^ǂ^	BETH	11.07	3.2E-4	0.002	down	1515-95-3
Amantadine	AMAN	12.61	0.001	0.008	down	768-94-5
Benzene, 1-(1,5-dimethyl-4-hexenyl)-4-methyl-	BEHX	16.45	0.002	0.019	down	644-30-4
Bergamotene	BERG	16.56	0.002	0.002	down	17699-05-7
**1,3,5-Undecatriene** ^**ǂ**^	**UNDE**	**11.9**	**0.002**	**3.7E-4**	**down**	**51447-08-6**
**Benzeneacetaldehyde** ^**ǂ**^	**BNAT**	**9.61**	**0.006**	**9.4E-4**	**down**	**122-78-1**
Sorbic acid vinyl ester	SORB	8.56	0.008	8.8E-4	down	42739-26-4
4(1 H)-Pyridone	4-PYR	8.43	0.010	0.019	up	108-96-3
**(E)-α-Bisabolene**	**CYCL**	**17.29**	**0.011**	**0.008**	**down**	**17627-44-0**
**Farnesene**	**FRNS**	**16.76**	**0.013**	**0.026**	**up**	**502-61-4**
Ethanone, 1-(1H-pyrrol-2-yl)-	ETHP	9.93	0.015	0.013	down	1072-83-9
Himachalol	HIMA	19.23	0.017	0.024	down	1891-45-8
2-Hexanone	2-HXO	4.43	0.017	0.009	down	591-78-6
Ethanone, 2-cyclohexyl-1-(1-methyl-1H-imidazol-4-yl)-	ETCH	19.51	0.019	0.003	down	69393-35-7
(Z)-γ-Bisabolene	1-MCY	16.94	0.021	0.005	down	495-62-5
1-(4-butoxy-2-methylphenyl)ethanone	4-BUT	19.42	0.021	0.004	down	NA
Benzenemethanol, 4-trimethyl-	BEME	11.97	0.022	0.014	down	1197-01-9
Benzaldehyde, 4-ethyl-	BENE	11.88	0.029	0.125	down	4748-78-1
Bisobolol	BIBO	19.22	0.034	0.072	down	515-69-5
Benzene, n-butyl-	BZNB	8.81	0.038	0.021	down	104-51-8
Benzene, [(methylsulfonyl)methyl]-	BNMS	15.08	0.045	0.026	down	3112-90-1
Benzene, 1,3-diethyl-5-methyl-	BNDI	12.75	0.050	0.050	up	2050-24-0
Formamide, N-phenyl-	FORM	12.54	0.051	0.022	down	103-70-8
**Benzaldehyde**	**BNZA**	**7.88**	**0.063**	**0.018**	**up**	**100-52-7**
**2-Propanamine, 2-methyl**	**2-PRO**	**2.55**	**0.069**	**0.582**	**down**	**75-64-9**
Cyclohexanol, 2,6-dimethyl-	CHXO	9.99	0.069	0.302	down	5337-72-4
1,4-Pentadiene	1-PEN	1.62	0.081	0.018	down	591-93-5
**D-Limonene**	**DLIM**	**9.32**	**0.084**	**0.066**	**down**	**5989-27-5**
Phenol, 2,4-dichloro-	PHEN	11.7	0.092	0.108	down	120-83-2
2-Pentanone, 3-methyl-	2-PTM	3.78	0.097	0.070	down	565-61-7
**Thiophene, 2-pentyl-**	**THIO**	**11.59**	**0.139**	**0.061**	**up**	**4861-58-9**
Benzene, 1-isothiocyanato-2-methyl-	BISO	13.73	0.156	0.029	up	614-69-7
**Hexadecane**	**HXDC**	**19.31**	**0.158**	**0.056**	**down**	**544-76-3**
Benzene, 1-ethenyl-4-ethyl	BNET	10.39	0.173	0.029	down	3454-07-7
β-Irone	3-BUT	19.05	0.210	0.094	down	79-70-9
Terpineol	TERP	12.07	0.226	0.043	down	98-55-5
(+)-α-himachalene	1-BEN	16.82	0.259	0.042	down	3853-83-6

Features bolded are also found in Table 2, VOCs that have an asterisk (*) were utilized for two class LDA, and VOCs with a cross (ǂ) were utilized for three class LDA. All VOCs in the table were utilized to discriminate between cancer/no cancer via PCA.

**Table 2:** List of the 30 VOCs that have a p-value less than 0.1 via the Student’s T-test or Wilcoxon’s Rank sum test when distinguishing between mice with localized and metastasized breast cancer.

Name	Abbrev.	RT (min)	T test p-value	Rank-sum p-value	Regulation	CAS #
Menadione*^,ǂ^	MENA	16.98	4.3E-4	7.1E-4	down	58-27-5
2,6-Dimethylhepta-2,4-diene*^,ǂ^	6-DMH	5.53	0.012	5.3E-4	up	4634-87-1
1-Octen-3-one*	1-OCT	8.26	0.040	0.136	down	4312-99-6
2,6,6-Trimethyl-2-cyclohexene-1,4-dione*	TRIM	11.32	0.050	0.009	down	1125-21-9
Bicyclo [2.2.1]heptane, 7,7-dimethyl-2-methylene* ^**,ǂ**^	**BCY2**	**8.52**	**0.069**	**0.072**	**up**	**471-84-1**
2(3H)-Furanone, 5-hexyldihydro-*	1-FUR	16.21	0.221	0.096	up	706-14-9
**Benzaldehyde**	**BNZA**	**7.88**	**0.003**	**0.002**	**down**	**100-52-7**
**D-Limonene**	**DLIM**	**9.32**	**0.005**	**0.018**	**up**	**5989-27-5**
**1,3,5-Undecatriene** ^**ǂ**^	**UNDE**	**11.9**	**0.007**	**0.007**	**up**	**51447-08-6**
**Benzene, 4-ethenyl- 1,2-dimethyl-**	**BNZE**	**10.45**	**0.009**	**0.020**	**up**	**27831-13-6**
2,6-Di-tert-butylbenzoquinone	DTBQ	16.24	0.012	0.016	up	719-22-2
**Hexadecane**	**HXDC**	**19.31**	**0.018**	**0.003**	**up**	**544-76-3**
5-methyl-2-propan-2-ylcyclohex-3-en-1-one	3-CON	13.99	0.037	0.037	down	NA
4-Hexen-3-one, 4,5-dimethyl	4-HEX	8.57	0.042	0.044	up	17325-90-5
**2-Propanamine, 2-methyl**	**2-PRO**	**2.55**	**0.050**	**0.044**	**down**	**75-64-9**
1H-Indole, 6-methyl-	1-IND	12.73	0.052	0.052	down	3420-02-8
Ethyl (E)-4-ethoxy-2-oxobut-3-enoate	ETOX	12.49	0.061	0.107	up	NA
Caryophyllene	CRYO	17.56	0.068	0.039	up	87-44-5
**(E)-α-Bisabolene**	**CYCL**	**17.29**	**0.070**	**0.097**	**up**	**17627-44-0**
2-Hexenal, 2-ethyl-	2-HEX	6.82	0.072	0.086	up	645-62-5
3-Heptanone	3-HEP	6.34	0.081	0.033	up	106-35-4
1-Propanone, 2-methyl-1-(2-methylphenyl)-	1-PRO	13.85	0.083	0.082	up	2040-14-4
**Farnesene**	**FRNS**	**16.76**	**0.088**	**0.748**	**down**	**502-61-4**
**Benzeneacetaldehyde** ^**ǂ**^	**BNAT**	**9.61**	**0.088**	**0.190**	**down**	**122-78-1**
**Pinocarvone** ^**ǂ**^	**PINC**	**11.66**	**0.092**	**0.132**	**down**	**30460-92-5**
n-Tridecan-1-ol	TRID	17.91	0.098	0.367	down	26248-42-0
2-Pentanone	2-PEN	2.8	0.099	0.025	up	107-87-9
**Thiophene, 2-pentyl-**	**THIO**	**11.59**	**0.197**	**0.058**	**up**	**4861-58-9**
Quinoline, 1,2,3,4-tetrahydro-	QUIN	13.19	0.326	0.051	down	635-46-1
2,4-Di-tert-butylphenol	DTBP	16.8	0.030	0.018	up	128-39-2

Features bolded are also found in Table 1, VOCs that have an asterisk (*) were utilized for two class LDA, and VOCs with a cross (ǂ) were utilized for three class LDA. All VOCs in the table were utilized to discriminate between localized/metastasized via PCA.

**Figure 2:**
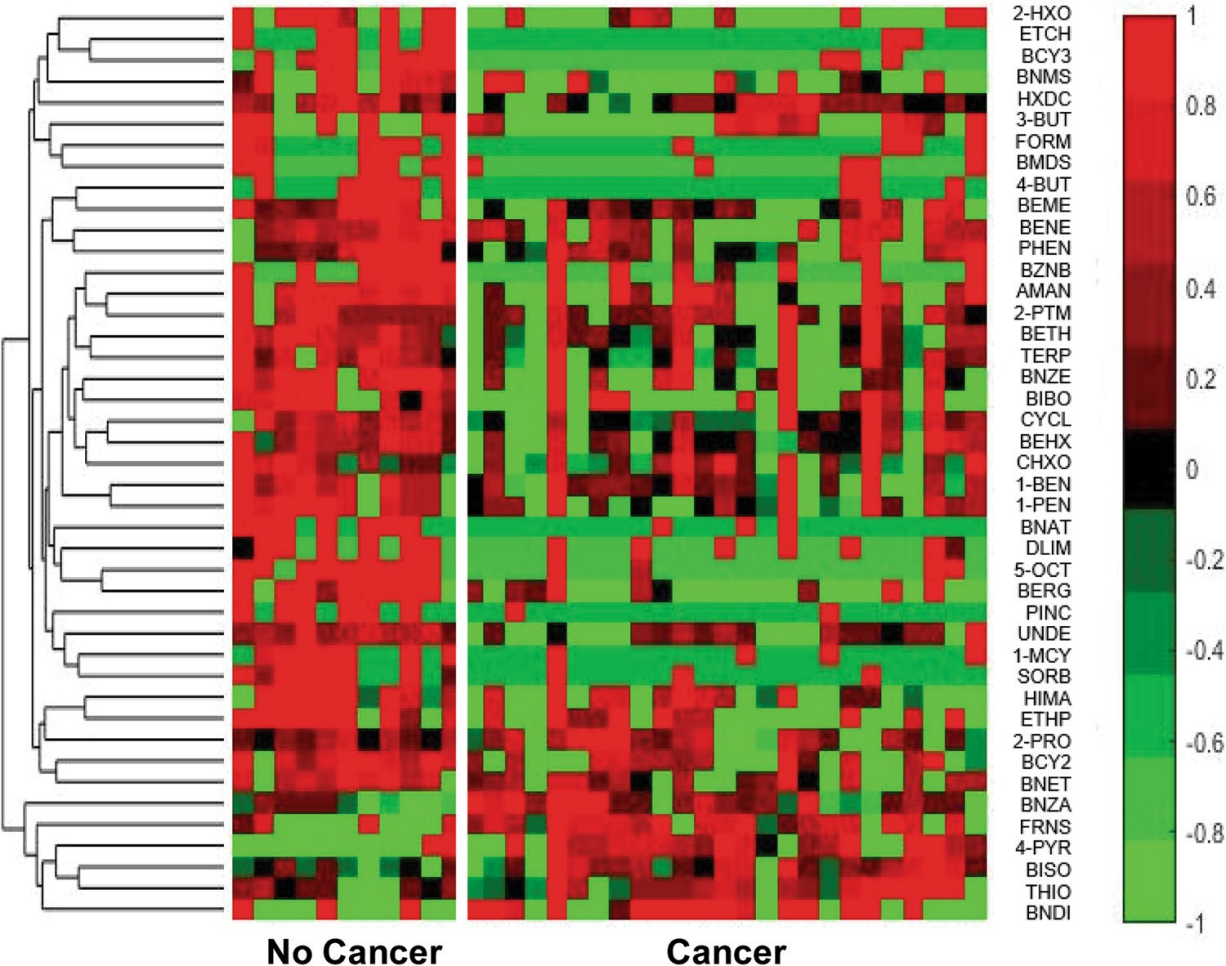
Hierarchical heatmap of the 43 VOCs (p-value < 0.1) different between mouse urine samples with and without breast cancer. Full compound names which are associated with the illustrated abbreviation can be observed in Table 1.

**Figure 3:**
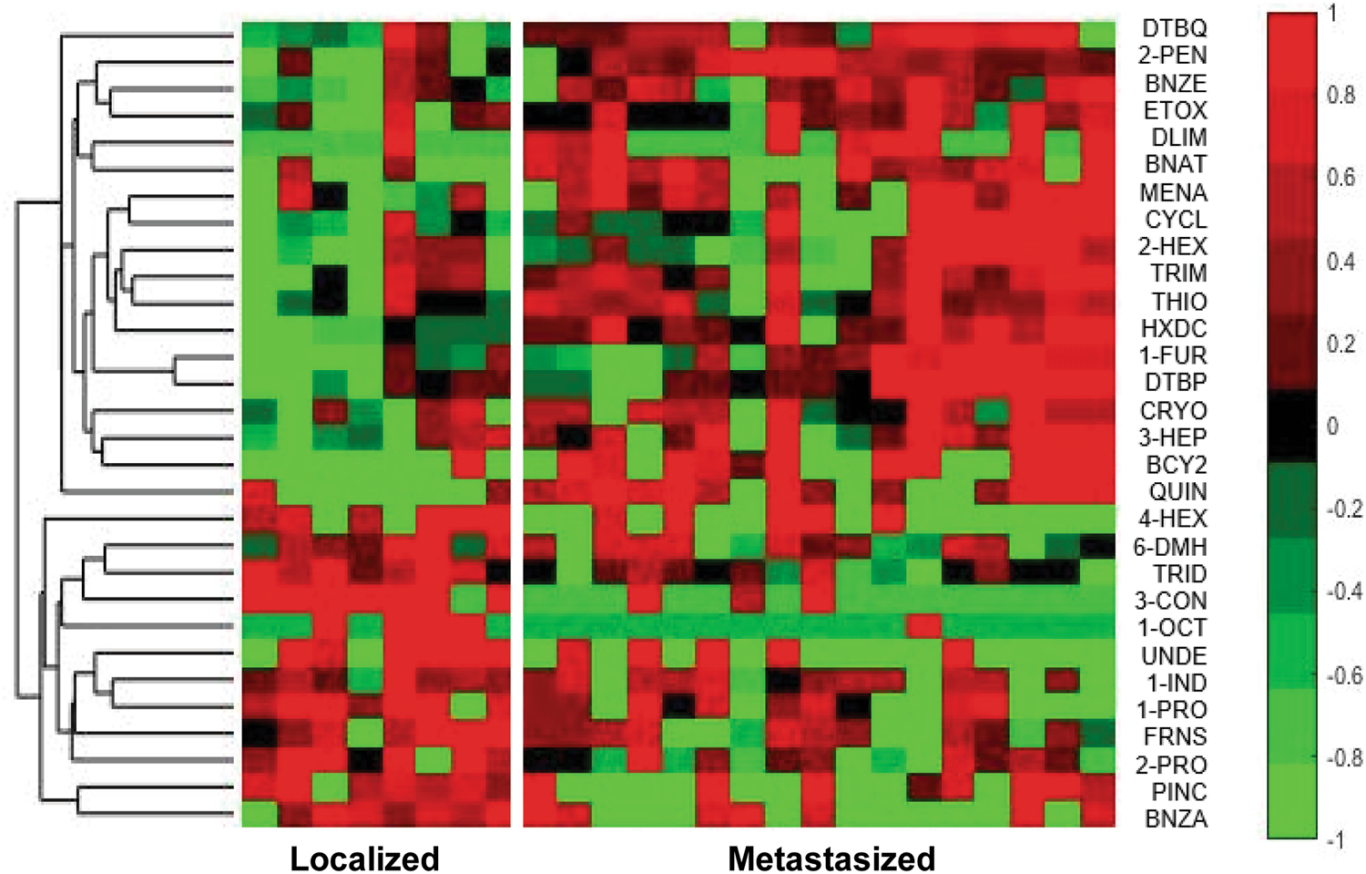
Hierarchical heatmap of the 30 VOCs that are (p-value < 0.1) different between mouse urine samples with localized breast cancer and metastasized breast cancer. Full compound names which are associated with the illustrated abbreviation can be observed in Table 2.

Table 1 shows all 43 features that univariately distinguish between mouse urine samples with and without breast cancer (p-value < 0.1), along with their associated retention times (RT), p-values, the CAS # and if the VOC is up or down regulated in breast cancer. Figure 2 illustrates a hierarchical heatmap of these 43 VOCs, where green illustrates a low concentration, red represents a relatively high concentration and black represents mean values (abbreviations used in Fig. 2 correspond to the full compound names in Table 1). For each VOC, there is a clear difference in concentration between the two classes of samples, and most of the VOCs are down regulated in mouse urine samples with breast cancer, and only six up regulated. Table 2 shows the 30 features differentiating metastatic breast cancer from localized breast cancer, and Fig. 3 shows a hierarchical heatmap of these 30 VOCs. From the identified VOCs for both comparisons (breast cancer/no cancer and localized breast cancer/metastatic), there are 12 VOCs that can be observed in both sets of data. The 12 common VOCs found in both data sets are bolded and can be observed in Tables 1 and 2.

Among these VOC biomarkers for both breast cancer and metastatic breast cancer, there is a wide range of size, structure and functionality. There are both commonalities and very slight differences in structure and function in these two different sets of potential metabolic biomarkers. Of the potential biomarkers for breast cancer, aromatic VOCs were the most common feature and non-conjugated cyclic compounds were the second most common structural feature. The third most frequently observed are ketones. VOCs that contain an ether or ester functional group are the least observed. The potential biomarkers for metastasized breast cancer have a similar distribution of functional groups. The three most frequently found structural features were again ketones, non-conjugated cyclic VOCs and aromatics. The three least frequently observed functional groups in the localized/metastasized data set are alcohols, esters and ethers. When compared to cancer/no cancer, sulfur-containing VOCs were less frequently occurring in the localized/metastasized data set. Also, there was one VOC that contained a chlorine atom in the cancer/no cancer set and there were none in the localized/metastasized group of VOCs.

### Multivariate statistical analysis

**Figure 4:**
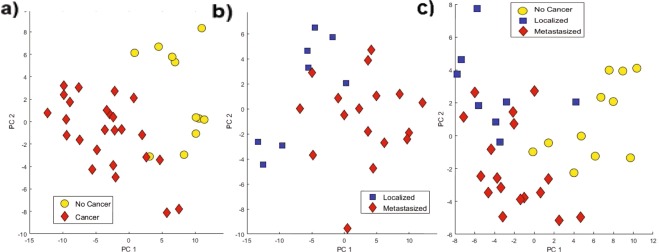
PCA utilizing (**a**) 43 VOCs to discriminate between mouse urine with and without breast cancer, (**b**) 30 VOCs to discriminate between mouse urine that was collected from mice that had cancer injected in the mammary pad (localized) and in the iliac artery (metastasized), (**c**) 20 VOCs to discriminate between mouse urine that was collected from all three classes (localized, metastasized and no cancer).

For both comparisons, PCA was executed utilizing all identified VOCs observed in Tables 1 and 2 (Fig. 4). When applied to samples with and without breast cancer, the first two principal component axes observed in Fig. 4(a) accounted for 35% of variation that exists between samples (PC 1–27%, PC 2–8%). When applied to the VOCs in the localized/metastasized data set, the first two principal components present in Fig. 4(b) accounted for 47% of variation between samples (PC 1–36%, PC 2–11%). PCA was also applied to the features that have potential discriminatory power to separate all three classes, and 20 VOCs with relatively low p-values resulted in the first two principal component axes accounting for 42% of variation between all samples (PC 1–31%, PC 2–11%) (Fig. 4(c)). All three representations show good distributions and an absence of outliers in the data sets.

**Figure 5:**
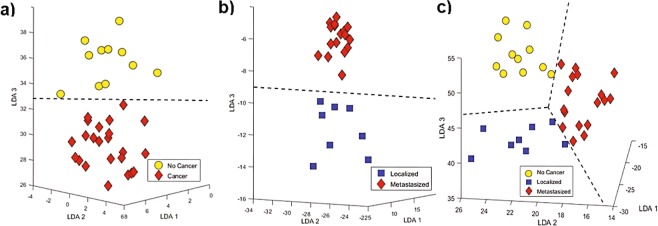
(**a**) LDA utilizing six VOCs to discriminate between mouse urine with and without breast cancer with 100% sensitivity and specificity, (**b**) LDA utilizing six different VOCs to discriminate between mouse urine that was collected from mice that had cancer injected in the mammary pad (localized) and mice that had cancer cells injected in the iliac artery (metastasized) with 100% sensitivity and specificity and (**c**) LDA using nine VOCs to perfectly discriminate between mouse urine that was collected from all three classes (localized, metastasized and no cancer).

Iterative LDA was applied to find a small set of VOCs with high classification accuracy. Six VOCs (the cancer panel) provided a perfect separation between all mice with and without breast cancer (Fig. 5(a) plots the samples along the principle linear discriminant axes, AUC = one on ROC curve not shown). The ROC curve for the five-fold cross validation results discriminating between cancer and no cancer gave an estimated AUC of 0.98 (95% confidence interval [0.85, 1.00]). The six VOCs that comprise the cancer panel are listed at the top of Table 1 and have an asterisk to note they have been utilized for multivariate analysis. Interestingly, all features were down regulated in the cancer samples and showed an absolute log 2-Fold Change more than 0.5 indicating a substantial decrease in concentration of these VOCs in urine for mice with breast cancer. Multicollinearity of the cancer panel was tested and found to be insignificant (VIF = 2.5). The cancer panel was further analyzed for overfitting by logistic regression. This test also showed perfect separation (AUC 5-fold cross validation = 0.97 (95% confidence interval [0.89, 1.00])).

For the case of localized compared with metastasized breast cancer samples, iterative LDA was applied to the 30 features that were listed in Table 2. Again, six compounds (the metastatic panel) gave a perfect separation of localized and metastasized mouse urine (Fig. 5(b)). Once again, five-fold cross validation was implemented and with cross validation, the AUC was 0.96 (95% confidence interval [0.75, 1.00]). The hierarchical heatmap in Fig. 3 and Table 2 demonstrate that these six metabolic VOCs in the metastatic panel are evenly distributed between up and down regulation in metastatic breast cancer. The VOCs are listed at the top of Table 2 and have an asterisk to note they comprise the metastatic panel. Multicollinearity of the metastatic panel was insignificant (VIF = 3.1). Logistic regression was also applied on the metastatic panel of VOCs and AUC was 0.94 (95% confidence interval [0.81, 1.00]). Finally, nine VOCs provided a perfect classification of all three sample classes via iterative LDA. Figure 5(c) plots the samples along the first three linear discriminant axes, and it can be observed there is a perfect classification of mice with no cancer, localized and metastasized breast cancer. However, this model showed evidence of being somewhat overfit as five-fold cross validation produced an overall correct detection rate of only 83%. The nine metabolic features are listed in Tables 1 and 2 and have a cross to note they have been utilized for multivariate analysis to distinguish between all three classes.

## Discussion

Volcano plots, in which statistical significance via the Student’s T-test is plotted against log 2-Fold Change between classes for all metabolites^39–41^, are useful for rapidly visualizing differences between up regulated and down regulated metabolites: Fig. 1 shows many more VOCs down regulated in breast cancer samples and there are more VOCs up regulated in metastasized breast cancer model relative to localized model, but to a lesser degree. This indicates that there is a more even distribution of metabolites that are up and down regulated in urine samples collected from mice with metastasized/localized breast cancer. This can be also seen in the hierarchical heatmaps in Figs 2 and 3. Benzaldehyde (BNZA) is the only labeled VOC present in both volcano plots and was observed to be up regulated in breast cancer and down regulated in metastatic breast cancer when compared to localized.

Univariate statistical analysis did not yield any VOC that could discriminate perfectly between cancer and no cancer samples or between metastatic and localized cancer. Therefore, multivariate analysis was utilized to identify a set of VOCs that could classify breast cancer samples from samples collected from mice with no cancer and metastatic samples from localized. PCA was implemented to visualize global patterns within the data set and to observe if any samples are outliers. Figure 4 shows the PCA distinguishing cancer/no cancer, localized/metastasized as well as localized/metastasized/no cancer, and there are no samples which are outliers. A supervised statistical analysis technique was implemented to increase the sensitivity and specificity for both classifications, as well as decrease the number of VOCs needed to separate sample classes via multivariate statistical analysis. LDA produces linear combinations of log2 integrated signal values from multiple VOCs to discriminate between two or more defined classes^42,43^. For each comparison, the top three features that could linearly discriminate between the two classes with the highest sensitivity and specificity values were generated. Next, one of the top three features were left out, and the next best three VOCs for classification were identified to produce a combination of four VOCs. A decision tree was utilized, where the best combinations were utilized to produce larger combinations of VOCs to further discriminate between sample classes for both comparisons. The decision tree was constructed until the result was inferior or perfect separation between classes was obtained.

The six compounds that distinguish both types of breast cancer from no cancer with 100% sensitivity and specificity via LDA in Fig. 5(a) (the cancer panel) are all down regulated in samples with cancer, showing the higher metabolic utilization of cancer compared to healthy mice. While an interesting finding, this result could be difficult to translate to clinical research where typically one looks for biomarkers up regulated by disease. A different set of six VOCs discriminated between localized and metastasized breast cancer via LDA in Fig. 5(b) (the metastatic panel) with three up regulated in metastatic and three up regulated in localized breast cancer. These VOCs are likely related to changes of the tumor local microenvironment. Bicyclo[2.2.1]heptane, 7,7-dimethyl-2-methylene (BCY2) was the only VOC that was found in both sets of 6 metabolites (cancer/no cancer and localized/metastasized). These two panels are not overfit because their average five-fold cross validation ROC values are relatively high (0.98 and 0.96 respectively) and when the Linear Discriminant function was perturbed with a Logistic Regression algorithm classifier, the AUC was still high (AUCs of 0.97 and 0.94, respectively)^22^. Even though there was only one VOC used in both sets of metabolites used to discriminate between cancer/no cancer and localized/metastasized, it displays there is possibly a set of VOCs that can be utilized to classify both data sets. A set of nine VOCs from both sets of data (Tables 1 and 2) perfectly distinguished between all three classes via LDA in Fig. 5(c).

There is a limited number of urinary biomarkers that were found in previous studies which analyzed VOCs in breast cancer cell lines. The VOCs that were found both in this study in Tables 1 and 2 and in breast cancer cell lines include: 3-heptanone, benzaldehyde, 2,4-di-tert-butylphenol and 2-pentanone. Other than the four VOCs found in both mouse urine and cell lines, there are many VOCs that share common structures and functionalities. One example of this was that  4-methyl-2-heptanone was discovered to be a biomarker in breast cancer cell lines, and  4,5-dimethyl-4-hexen-3-one was found to be a biomarker for breast cancer and metastatic breast cancer in mouse urine^3,15^. Interestingly, the mouse urine contained more unsaturated compounds than the breast cancer cell lines. Even though there were not many VOCs that were detected as potential biomarkers for breast cancer in mouse urine that were also observed in breast cancer cell lines, it still gives confirmation that some of the VOCs present in urine that change significantly are due to changes in the tumor itself. Since many metabolic VOC biomarkers for metastatic breast cancer were not observed in cell lines, many biomarkers detected in mouse urine may be changing concentration due to interactions of the tumor cells and the local microenvironment. There were also a small set of potential urinary biomarkers for breast cancer found in this study that were found in biological breath in humans with breast cancer^10,11^. 1,4-pentadiene, D-limonene and 2,6 di-tert-butylbenzoquinone were found in both human breath and mouse urine as potential biomarkers for breast cancer. Again, even though there were a limited number of common VOCs, there were many similarities in structure between the sets of VOCs. Many aromatic VOCs and ketones were found in biological breath and mouse urine to be potential volatile markers of breast cancer^10–13^. Finally, it is noted that one study has reported VOCs from human urine, comparing women with invasive breast cancer with controls (largely men) with no cancer^14^. Their analysis utilized acidified samples which highlight different VOC types than pH neutral or basic samples^15^, and they analyzed only invasive cancer, so their results and ours would not be expected to be the same.

Many of the potential biomarkers for breast cancer are involved in the biosynthesis of terpenoids; these VOCs include bicyclo[2.2.1]heptane, 7,7-dimethyl-2-methylene, farnesene, caryophyllene, D-limonene, pinocarvone, himachalol, himachalene, bisabolol, bisabolene and other VOCs in Table 1. Terpenes and terpenoids have an antioxidant and therapeutic effect on cancerous tumor cells^44^, which is fascinating because they were largely depleted in the samples with cancer. This study employed a simplified model for comparing localized and metastatic breast cancer in which the same tumor cells are injected into different sites (mammary pad versus iliac artery). The first result was that a panel of 6 VOCs can be used to classify whether mice had either form of cancer: the test gave a perfect separation using either of two classification models, LDA or logistic regression, with high values for cross validation/CI testing. Further, the study identified a separate metastatic panel that was able to classify tumor location perfectly via LDA or logistic regression. This study shows that not only do VOCs change due to an alteration in metabolism (cancer/no cancer model), but it also shows unique VOCs released by specific tumor – microenvironment interactions (localized/metastasized model). This study demonstrates the potential of volatile metabolomics to identify biological markers tied to breast cancer. One limitation is the study was carried out in a controlled environment on immune-compromised mice. While greater metabolic heterogeneity will be present in human samples, the same or similar biomarkers likely can be used to better explore and understand tumor/microenvironment interactions in humans. Similar metabolic biomarkers found in human urine can inspire the development of an inexpensive, accurate and noninvasive biological assay for breast cancer.

## Data Availability

The authors provide no restriction on the availability of methods, protocols, instrumentation and data utilized in the following article. All data will be available from the corresponding author by request.
